# Association between Dietary Fatty Acid Intake and Liver Steatosis and Fibrosis in a Sample of Mexican-Origin Hispanic Adults with Overweight or Obesity

**DOI:** 10.3390/ijerph20043103

**Published:** 2023-02-10

**Authors:** Melissa Lopez-Pentecost, Brian Hallmark, Cynthia A. Thomson, Floyd Chilton, David O. Garcia

**Affiliations:** 1 Sylvester Comprehensive Cancer Center, University of Miami Miller School of Medicine, Miami, FL 33136, USA; 2 The BIO5 Institute, University of Arizona, Tucson, AZ 85721, USA; 3 Department of Health Promotion Sciences Tucson, University of Arizona, Tucson, AZ 85721, USA; 4 Department of Nutritional Sciences, University of Arizona, Tucson, AZ 85721, USA

**Keywords:** fatty acid, steatosis, fibrosis, NAFLD, Mexican-origin Hispanics

## Abstract

Rates of non-alcoholic fatty liver disease (NAFLD) vary dramatically among Hispanic subpopulations, with Mexican-origin (MO) Hispanics experiencing a disproportionate burden. This study examined dietary fatty acid (FA) intake among overweight and obese MO Hispanic adults in the United States (US) and evaluated its association with liver steatosis and fibrosis. Participants (N = 285, MO Hispanic adults) completed 24-h dietary recalls to assess dietary FA exposure. Liver steatosis and fibrosis were estimated using transient elastography (FibroScan^®^). Multiple regression analysis tested relationships between FA intakes and liver steatosis or fibrosis, adjusting for age, sex, body mass index (BMI) and total energy. A total of 51% (n = 145) of participants were suspected to have NAFLD and 20% self-reported a type 2 diabetes diagnosis. No significant association was observed between Linoleic Acid and α-Linolenic Acid (LA:ALA) ratio, or omega-6 to omega-3 (n-6:n-3) ratio and liver steatosis. However, a one-point increase in the LA:ALA ratio resulted in a 1.01% increase in the liver fibrosis scores (95% CI: [1.00, 1.03]; *p* = 0.03), and a one-point increase in the n-6:n-3 ratio resulted in a 1.02% increase in liver fibrosis score (95% CI: [1.01, 1.03]; *p* = 0.01). Further research is needed to determine if modulation of FA intake could reduce NAFLD risk in this high-risk population.

## 1. Introduction

Hispanics of Mexican origin (MO) are the largest subpopulation in the United States (U.S.) and account for nearly two-thirds of the Hispanic population [1]. MO Hispanics have among the highest rates of metabolic conditions that are strongly associated with non-alcoholic fatty liver disease (NAFLD) including obesity and type 2 diabetes [2]. In fact, rates of NAFLD vary dramatically among Hispanic subpopulations, with MO Hispanics experiencing a disproportionate (33%) burden compared with Hispanics of Dominican (16%) or Puerto Rican origin (18%) [3]. Nearly 30% of individuals with NAFLD are at increased risk of developing cirrhosis, portal hypertension and hepatocellular carcinoma (HCC). HCC is projected to become the leading cause of liver-related morbidity and mortality in the U.S. [4]. Lifestyle changes, including improvements in diet quality and increased physical activity, are prevention and treatment strategies recommended for NAFLD [5].

Nutritional components hypothesized to impact NAFLD development include an imbalance in the consumption of omega-6 (n-6) relative to omega-3 (n-3) polyunsaturated fatty acids (PUFAs) that resulted from a marked increase in n-6 PUFA containing cooking oils and other processed foods in the past century [6,7,8]. Ratios of dietary n-6 to n-3 have been reported to be ~10:1 in most western populations; this is significantly higher than the 1:1 to 4:1 ratios reported to have health benefits [9,10]. This dietary shift has been observed in MO Hispanics, largely as a result of immigration and increasing acculturation to westernized diets of the U.S. [11,12]. Specifically, moving away from a *traditional Mexican* dietary pattern, shown to improve metabolic health [13,14,15,16], to a western dietary pattern associated with increased NAFLD risk and other obesity-related diseases, has been proposed to have devastating health effects and contribute to health disparities observed among MO Hispanics [17,18].

The vast majority of ingested PUFAs are n-6 and n-3 18-carbon (18C-) PUFAs such as linoleic acid (LA; 18:2, n-6) and α-linolenic acid (ALA, 18:3, n-3). These in turn compete in a biosynthetic pathway during conversion to biologically active highly unsaturated fatty acids (HUFAs) such as arachidonic acid (ARA; 20:4, n-6), eicosapentaenoic acid (EPA; 20:5, n-3) and docosahexaenoic acid (DHA; 22:6, n-3). ARA and its oxylipin metabolites including eicosanoids such as prostaglandins, thromboxanes, and leukotrienes, typically have pro-inflammatory and pro-coagulant properties, while n-3 HUFAs (EPA and DHA) and their metabolites have anti-inflammatory and pro-resolving activities [19,20]. Consequently, the levels and ratios of dietary 18C-PUFAs that enter this pathway dictate the balance of n-6 to n-3 HUFAs that are synthesized, and the dramatic shifts in 18C-PUFA ratios discussed above have great capacity to alter the balance of n-6 and n-3 HUFAs and their metabolites. This imbalance may be further exacerbated by the fact that MO Hispanics have high frequencies of ancestral fatty acid desaturase (*FADS*) variants that potentially limit their capacity to produce HUFAs and particularly n-3 HUFAs [21]. This gene by diet interaction may be linked to an observed depletion in n-3 HUFA levels observed among patients with NAFLD [22,23]. Given these genetic and dietary factors, the MO population may benefit from dietary interventions that focus on n-3 HUFA consumption or supplementation to reduce the imbalance of n-6 and n-3 HUFAs and their metabolites and thus prevent NAFLD progression.

To date, little information is known about the current intake of fatty acids (FAs) among MO Hispanics, despite the elevated risks for NAFLD and adverse HCC outcomes. The objective of the present study was to describe the intake of dietary fat, saturated FAs, monounsaturated FAs and n-6 and n-3 PUFAs among MO Hispanics, and to evaluate potential associations of these fats with liver steatosis and fibrosis in this population. This information addresses a vital gap in in the literature that will serve to inform the design and development of future dietary interventions to address the NAFLD burden in MO Hispanics.

## 2. Methods

### 2.1. Participants and Procedures

The study sample for this analysis was derived from a previous observational cross-sectional study of MO men and women in Tucson, AZ [24]. Participants were eligible to participate in this study if they self-identified as being of Mexican origin and were between the ages of 18 and 64. Additionally, given excess body weight (BMI) is a risk factor for NAFLD and the primary study targeted high-risk individuals only, eligibility criteria were limited to those with BMI ≥ 25 kg/m^2^. Participants were asked to self-report any comorbid conditions, including type 2 diabetes, that were required to be under control at the time of the study. Participants with excessive alcohol intake, as defined as more than 21 standard drinks per week for men and 14 drinks per week for women, were not eligible to participate in the study [5]. A total of 285 (n = 103 men, 182 women) participants were successfully recruited and completed the study. Participants attended a single in-person visit to a specialized liver clinic where data were collected using standardized protocols. Participants were given a variety of study questionnaires to complete in English or Spanish based on their language of preference. The study protocol was approved by the Institutional Review Board (IRB) (IRB #1902380787) and all participants provided written informed consent prior to enrolling in the study.

### 2.2. Liver Assessment and NAFLD

Liver steatosis and fibrosis were measured by a trained physician or technician using transient elastography (TE) (FibroScan^®^). TE measures liver fat infiltration (steatosis) as controlled attenuation parameter (CAP) scores in units of dB/m and liver stiffness (a validated proxy for fibrosis [25,26]) expressed as kilopascals (kPa). A CAP score cutoff of 288 dB/m, equivalent to 5% hepatic steatosis, was used to determine the presence of suspected NAFLD based on the recommendation from Caussy et al., given its relevancy to our study population [25]. Liver fibrosis measurements can range from 1.5 kPa to 75 kPa, with higher values indicating more severe fibrosis and values of 7.9 kPa or greater indicating significant fibrosis [26]. Cut-off fibrosis severity values were selected based on previous literature as follows; <7.9 kPa (F0-F1), 7.9 to <8.8 kPa (F2), 8.8 to <11.7 kPa (F3), and ≥11.7 kPa (F4) [26]. Participants were asked to fast for at least three hours before their liver ultrasound. Measurements were obtained at a single time point during the participant’s clinical study and a minimum of 10 measurements were taken from each participant using an established CAP interquartile range of <30 dB/m for quality assurance across measurements [25,27].

### 2.3. Dietary Intake Assessment

Dietary intake was assessed through 24-h dietary recalls using the United States Department of Agriculture multiple-pass method [28]. For each participant, three recalls were conducted over a two-week period taking place on three non-sequential days including two weekdays and one weekend day. Initiation of the dietary recalls was within a week following the participant’s study visit. All 285 participants completed all three separate dietary recalls on different days of the week to account for variability in dietary habits. Participants were provided a food amounts booklet to facilitate reporting of portions and amounts. All 24-h recalls were administered by trained, bilingual and bicultural personnel familiar with the diets of MO Hispanics in the region, via telephone using validated protocols. Dietary data were processed using the Nutrition Data System for Research (NDSR-2019). Primary outcome variables for this analysis included total intake of dietary fat, saturated fat, MUFA, PUFA, and n-6 to n-3 PUFA ratios.

## 3. Statistical Analysis

For each participant, intake values for each nutrient were computed as the mean of the three individual dietary recalls. Histograms of intakes were investigated and, in some cases, data were log-transformed due to skewedness. Additional values of interest, for example, the LA to ALA ratio and the n-6 to n-3 ratio, were computed from the individual level nutrient intake data. The (possibly transformed) intake values were compared between suspected NAFLD cases and controls using *t*-tests. Multiple regression was used to examine relationships between nutrient intakes (as % of total) or ratios and liver steatosis or fibrosis scores. Three models were conducted; crude unadjusted (model 1), adjusted for age, sex, BMI, and total energy intake (kcals) as covariates (model 2), and covariates shown to have a significant interaction in a stepwise regression analysis, which included sex and BMI only (model 3). *p*-values were not adjusted. All statistical computations were performed with R 4.0.

## 4. Results

### 4.1. Sample Characteristics

**Figure 1:**
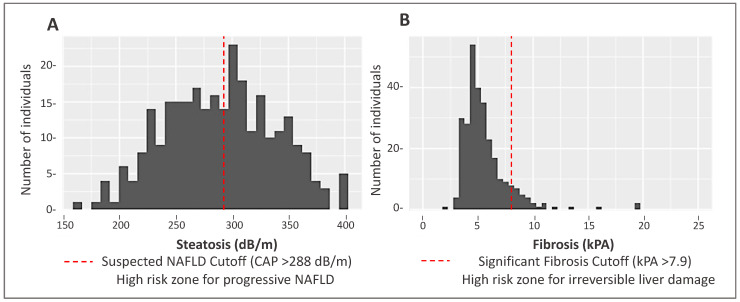
Distribution of steatosis and fibrosis scores as measured by transient elastography (FibroScan^®^) in a Mexican-origin Hispanic sample from Southern Arizona (N = 285). (**A**) Distribution of steatosis CAP scores (Mean: 289.5 dB/m, Standard Deviation [SD]: 48.9 dB/m). (**B**) Distribution of liver fibrosis values (Mean: 55.67 kPa, SD: 2.83 kPa).

Demographic and clinical data for MO Hispanic participants (n = 285) are presented in Table 1. Participants were on average 45 years of age and 64% were women. In total, 145 participants (51%) had a CAP score ≥ 288 dB/m indicating the suspected presence of NAFLD. Significant differences were observed for several characteristics between non-NAFLD and suspected NAFLD cases. Specifically, suspected NAFLD participants had a higher proportion of individuals with obesity and type 2 diabetes. The distribution of liver steatosis and fibrosis scores are shown in Figure 1.

### 4.2. Dietary Intake

Dietary intake values by suspected NAFLD status are presented in Table 2. Overall mean caloric intake in our study sample was 1492 ± 539 kcal/day. After adjustment for multiple testing, there were no statistically significant differences between groups for total caloric intake (95% CI: −13.8, 237; *p* = 0.08), and percent calories from carbohydrates (95% CI −0.18, 3.77; *p* = 0.16). However, statistically significant differences were observed between the groups for total carbohydrate and sugar intake with the suspected NAFLD group consuming 19.2 g (95% CI: [3.01, 35.3]; *p* = 0.02) and 7.4 g ([−0.89, 15.8]; *p* = 0.02) lower, respectively, than the non-NAFLD group. Given the alcohol exclusion criteria for the original study, alcohol consumption was overall low with a mean consumption of 1.65 ± 4.9 g, among the suspected NAFLD group consuming approximately half the consumption compared to non-NAFLD participants. Intakes of other nutrients did not appear to vary by NAFLD status.

### 4.3. Fat Intake

Overall, approximately 35% of calories came from fat and total fat intake averaged 59.6 g/day; 54.1 g/day for women and 69.4 g/day for men. Approximately 10–15% of daily calories came from saturated fatty acid (SFA), another 10–15% from monounsaturated fatty acid (MUFA), and an additional 5–10% of energy from PUFAs (Table 2). The primary SFA were palmitic acid (C16:0), stearic acid (C18:0), and myristic acid (C14:0). Oleic acid (C18:1, n-9) was the primary MUFA (90% of the total) and the fatty acid with the highest intake overall (~20 g/day).

### 4.4. High n-6 to n-3 Ratio

**Figure 2:**
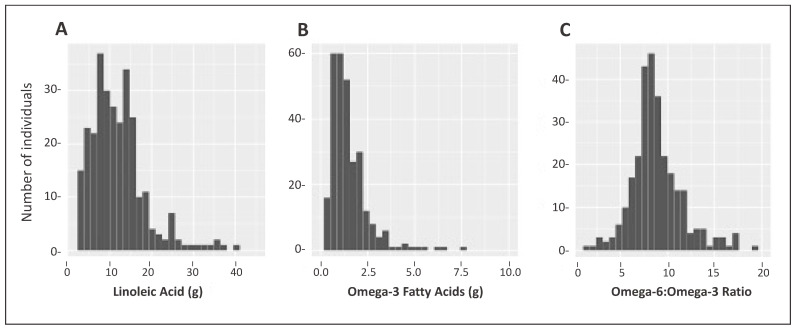
Intake distribution of linoleic acid (g), omega-3 fatty acids, (g) and Omega-6 to Omega-3 ratio in a Mexican-origin Hispanic sample from Southern Arizona (N = 285). (**A**) Distribution of Omega-6 (predominantly Linoleic Acid); (**B**) Distribution of Omega-3 (α-Linolenic Acid, Eicosapentaenoic Acid, Docosapentaenoic Acid, and Docosahexaenoic Acid); (**C**) Distribution of Omega-6 to Omega-3 ratio. Note the difference in the scales for both axes.

The majority of PUFA intake was n-6 fatty acid LA; C18:2, with a mean intake of 12 ± 6.5 g/day, accounting for 6.9 ± 2.2% of total energy (Table 2). Figure 2A shows the distribution of LA intake. In contrast, relative to n-6, n-3 fatty acid intake was overall very low, with the majority consisting of ALA 1.50 g/day ± 1.01 accounting for 0.82 ± 0.37% of total energy, with low intakes of EPA and DHA (both less than 0.1 g/day on average). The mean n-6 to n-3 PUFA ratio for the non-NAFLD group was 8.95 ± 2.65 compared to 8.71 ± 2.70 (*p* = 0.44) for suspected-NAFLD cases. The distribution of all n-3s combined is plotted in Figure 2B. These intakes resulted in an average n-6 to n-3 ratio of 8.9 (range: 2.46–19.8), with only nine subjects having ratios less than 5 (Figure 2C).

### 4.5. Fatty Acid Intake and Liver Steatosis and Fibrosis

Interaction analysis showed BMI was strongly associated with both steatosis and fibrosis, explaining 17.6% (*p* < 0.001) and 11.7% (*p* < 0.001) of their variation, respectively. For this reason, regression models 2 and 3 included BMI as a confounder. Overall, none of the nutrient intakes (as % of total) explained more than 2% of the total variation (R^2^) in steatosis or fibrosis scores and the regression F-tests were not significant (i.e., all *p* > 0.05).

Our results show the LA:ALA ratio was not statistically significantly associated with liver steatosis in Model 1-crude (*p* = 0.87) nor Model 2 (*p* = 0.83). Similarly, the n-6:n-3 ratios were not statistically associated with liver steatosis in either models (Model 1: Estimate = −0.51, *p* = 0.64; Model 2: Estimate = −0.11, *p* = 0.91). Stepwise regression for steatosis yielded no significant covariates (as in the crude model); therefore, we do not report results for Model 3. Our results also indicate that both the LA:ALA ratio and the n-6:n-3 ratio were positively associated with liver fibrosis in both Model 2, which adjusts for literature-derived covariates known to influence NAFLD risk, and Model 3, which includes covariates with a significant interaction in a stepwise regression analysis (Table 3). In particular, a one-point increase in the LA:ALA ratio resulted in a 1.01% increase in liver fibrosis scores (95% CI: [1.00, 1.03]; *p* = 0.03), and a one-point increase in the n-6:n-3 resulted in a 1.02% increase in liver fibrosis score (95% CI: [1.01, 1.03]; *p* = 0.01).

## 5. Discussion

The present study sought to describe dietary fatty acid intake among MO Hispanics in Southern Arizona and to determine whether intakes were associated with suspected NAFLD status, particularly liver steatosis and fibrosis. Findings from this work show there was no significant association between fatty acid intake and liver steatosis. However, ratios of LA:ALA and n-6:n-3 were both found to be significantly associated with liver fibrosis. To our knowledge, this is the first study to investigate these associations in a sample of exclusively MO Hispanic participants.

This study was a secondary analysis of an observational study in MO Hispanics who were overweight or obese. Over half of participants (51%) were suspected of having NAFLD based on transient elastography and 20% self-reported a type 2 diabetes diagnosis. Research has shown that these obesity-related metabolic diseases are highly interconnected and engage in a positive feedback loop where NAFLD increases risk of type 2 diabetes and diabetes exacerbates severity of NAFLD that can lead to more serious conditions such as cirrhosis and HCC [29]. Interestingly, we found that a higher proportion of suspected NAFLD cases were women (66%) compared to men (34%). This observation could potentially be explained by the fact that a higher proportion of women had obesity (69% vs. 58%) and were carriers of at least one PNPLA3 risk allele (66% vs. 34%). Previous studies indicate that carriers of the risk allele show > 2-fold higher levels of hepatic fat than non-carriers [30]. Therefore, this finding highlights the intricate relationship of various factors that play a role in the development and severity of NAFLD that should be taken into consideration in future efforts seeking to address alarming NAFLD rates.

Similar to previous findings with PUFAs [31], LA intake accounted for the majority of n-6 PUFA consumption and ALA was the most consumed n-3 PUFA. Intake of n-3 HUFAs, both EPA and DHA, were very low in this population. The mean n-6:n-3 FA ratio of 8.9 (range: 2.46–19.8) reported in the current study is substantially lower than the ratio of 16.0 found by Ramirez-Silva in another Mexican sample [31]. The ratios n-6:n-3 PUFAs in the current study were similar to the compositions and ratios observed throughout the U.S. and to a western diet pattern [9,10,32].

Findings in this study differ from those in the literature. It is important to highlight that previous studies have mainly focused on n-3 intake and supplementation. Additionally, such studies have included populations other than MO Hispanics and different outcome measures of liver disease; therefore, direct comparison across study findings are limited. In our study, no associations were observed between dietary intake of n-3, or the ratio of n-6:n-3 and liver steatosis. In contrast, numerous clinical trials have shown association between n-3 HUFAs and improvement of liver steatosis and/or other markers of NAFLD [33,34,35]. Congruent results were observed in one study investigating fatty acid intake ratios and disease severity in NAFLD patients where no associations were observed [36] as well as in a controlled study testing high fat diets differing in n-6:n-3 ratios in a Maurine model [37]. While findings for FA intake ratios and steatosis were null in our study, significant associations were found between FA intake ratio and liver fibrosis, which are congruent with others in the literature. Specifically, in the current analysis, significant association between n-6:n-3 PUFA ratios and liver fibrosis scores was observed, such that a greater n-6:n-3 ratio corresponded to a significantly higher percentage of fibrosis scores. Such results are in line with several in the literature where supplementation of n-3 intake, and subsequent reduction in n-6:n-3 ratio, was associated with improvement in hepatic fibrosis [38]. On the other hand, recent studies have shown no association between n-3 intake and any histological feature of NASH [39] highlighting the need for more robust evaluation of this association. Although it was not feasible to confirm NAFLD diagnosis in the current study, results show similar associations to studies where NAFLD cases were confirmed. Araya et al., 2004, tested the hypothesis that depletion of hepatic HUFAs is a major mechanism contributing to the pathogenesis of fatty liver in NAFLD patients and showed that higher n-6:n-3 ratios within the liver of NAFLD patients caused a derangement in the capacity of the liver to regulate lipid metabolism [23]. Han et al., 2014, reported that low levels of dietary n-3 PUFAs were associated with elevated NAFLD in Korean men (OR: 2.16; *p*-trend = 0.030) [32]. Collectively, these findings point out the need for further studies to evaluate the effects of n-6 and n-3 intakes on liver steatosis and fibrosis. In particular, these studies emphasize a critical need to investigate the effects of n-6 and n-3 PUFA in diverse populations such as MO Hispanics where metabolic responses to dietary PUFAs may vary.

Differences in other non-lipid dietary factors, such as carbohydrate and sugar intake, were observed in suspected NAFLD cases versus non-NAFLD participants. Carbohydrate intake, particularly, sugar in the form fructose, has been reported to stimulate the development of NAFLD [40]. However, our study results show that participants in the suspected NAFLD group had a statistically significant lower intake of total carbohydrate and total sugars compared to participants in the non-NAFLD group. While robust efforts were undertaken by the research team to address commonly known issues with dietary reporting, it is suspected that this played a role in our current findings. Participants in the suspected NAFLD group were overall more obese and more likely to have diabetes compared to the non-NAFLD group. In addition, the suspected NAFLD group had a larger proportion of women. Previous studies have reported that such sociodemographic characteristics are associated with dietary underreporting [41,42]. Specifically, underreporting of carbohydrate intake is particularly relevant among Spanish-speaking Hispanic women [43]. Underreporting sugar could also be related to the large number of individuals with diabetes among this group, many of whom know sugar consumption is recommended to be limited for the management of this disease.

While there are strengths in our study including the study sample of specifically MO U.S. Hispanic adults, measured liver steatosis and fibrosis estimates, representation across sexes, and data collection (including repeat 24-h recalls) in the language of preference, several limitations also exist. First, our study sample was composed of only individuals with overweight or obesity (BMI ≥ 25 kg/m^2^) and therefore, the generalizability of our findings should not be extended to those who do not meet this clinical criterion. It is also well recognized that increased plasma fatty acid levels are elevated in obesity due to numerous factors [44]. Such high levels of endogenous circulating fatty acids may limit the impact of dietary fatty acids on total exposure. This work should expand to normal BMI individuals to determine if dietary exposure and elevated circulating FAs drive an at-risk phenotype of normal BMI individuals. Second, while the 24-h recalls were administered by trained, bilingual and bicultural personnel familiar with the diets of MO Hispanics in the region using validated protocols, there is a need to continue to improve on dietary assessment measures for diverse populations such as MO Hispanics in order to overcome existing dietary misreporting. Importantly, future studies focused on understanding the association of dietary fatty acid exposure and the levels of circulating fatty acids in MO Hispanics are important next steps in understanding the overall impact of circulating levels of fatty acids. Lastly, measurements of liver steatosis and fibrosis were obtained using transient elastography (FibroScan^®^), which, while robust, is not the gold standard and therefore MRI confirmation of disease severity is warranted.

## 6. Conclusions

Our findings indicate that the dietary n-6:n-3 PUFA ratio is associated with liver fibrosis in a sample of MO Hispanic adults with overweight or obesity. Dietary n-6 and n-3 PUFA intakes were comparable to what is observed in general U.S. populations and were not associated with liver steatosis. Additional studies are warranted to include large samples, normal weight individuals and even more robust and serial measures of liver pathology to determine whether modulation of fatty acid intake is an appropriate dietary strategy to reduce NAFLD risk in this high-risk population.

## Figures and Tables

**Table 1:** Demographic and clinical characteristics of a Mexican-origin Hispanic sample from Southern Arizona by suspected NAFLD status (N = 285).

Characteristic	Non-NAFLD Participants (n = 140)	Suspected NAFLD Cases ^a^ (n = 145)
Liver steatosis (CAP dB/m, mean ± SD) *	246.61 ± 30.25	328.86 ± 28.80
Liver fibrosis (kPa, mean ± SD) *	4.93 ± 1.20	6.39 ± 3.66
Age (years, mean ± SD)	44.5 ± 11.6	44.8 ± 10.8
Sex, n (%)		
Men	53 (38%)	50 (34%)
Women	87 (62%)	95 (66%)
Health insurance, n (%)		
Yes	90 (64%)	85 (59%)
Education, n (%)		
Less than high school	34 (24%)	46 (32%)
High school or GED	30 (21%)	35 (24%)
Greater than high school	76 (54%)	64 (44%)
Language Spoken at Home, n (%)		
English	40 (29%)	36 (25%)
Spanish	100 (71%)	109 (75%)
Birthplace, n (%)		
Foreign Born	92 (66%)	107 (74%)
U.S. Born	48 (38%)	38 (26%)
BMI (kg/m^2^, mean ± SD)	30.7 ± 4.1	34.5 ± 5.6
BMI Classification, n (%) *		
Overweight (25–29.9 kg/m^2^)	69 (49%)	33 (23%)
Obese (≥30 kg/m^2^)	71 (51%)	112 (77%)
Type 2 Diabetes, n (%) *	11 (8%)	17 (12%)
Cancer History, n (%)	2 (1%)	2 (1%)

^a^ Participants categorized as suspected NAFLD cases if CAP score was ≥288 dB/m * Indicates statistically significant differences between groups with *p*-value < 0.01

**Table 2:** Dietary intake values by suspected NAFLD Status in a Mexican-origin Hispanic sample from Southern Arizona (N = 285).

	Non-NAFLD Participants (n = 140)		Suspected NAFLD Cases (n = 145)	
Component	Mean	SD	Mean	SD
Total Grams	3581.66	1450.34	3596.22	1347.85
Energy (kcal)	1548.45	552.81	1436.75	522.34
Total Protein (g)	69.30	26.26	65.83	25.79
Total Carbohydrate (g) *	184.82	70.92	165.64	67.51
Total Sugars (g) *	73.18	35.29	65.74	36.10
Added Sugars (g)	46.05	33.36	41.12	31.30
Alcohol (g) *	2.27	5.58	1.05	3.99
Cholesterol (mg)	280.20	153.09	292.12	152.02
Total Fat (g)	60.37	26.24	58.86	26.83
Total Saturated Fatty acid (SFA) (g)	19.11	9.07	18.73	9.72
SFA 14:0 (myristic acid) (g)	1.46	0.89	1.49	1.16
SFA 16:0 (palmitic acid) (g)	10.76	4.75	10.63	5.15
SFA 18:0 (stearic acid) (g)	4.71	2.43	4.52	2.39
Total Monounsaturated Fatty acid (MUFA) (g)	21.54	9.55	21.25	10.35
MUFA 16:1 (palmitoleic acid) (g)	0.97	0.50	1.01	0.60
MUFA 18:1 (oleic acid) (g)	20.13	8.94	19.78	9.62
Total Polyunsaturated Fatty acid (PUFA) (g)	14.15	7.63	13.46	7.29
PUFA 18:2 n-6 (linoleic acid) (g)	12.36	6.69	11.65	6.30
PUFA 18:3 n-3 (alpha-linolenic acid [ALA]) (g)	1.38	0.89	1.34	0.95
PUFA 20:4 n-6 (arachidonic acid) (g)	0.14	0.08	0.16	0.23
PUFA 20:5 n-3 (eicosapentaenoic acid [EPA]) (g)	0.03	0.04	0.04	0.14
PUFA 22:5 n-3 (docosapentaenoic acid [DPA]) (g)	0.02	0.02	0.02	0.04
PUFA 22:6 n-3 (docosahexaenoic acid [DHA]) (g)	0.07	0.11	0.09	0.27
Total Trans-Fatty acid (TRANS) (g)	1.50	0.97	1.49	1.08
n-3 Fatty acid (g)	1.50	0.95	1.51	1.08
n-6: n-3 ratio	8.95	2.65	8.71	2.70
LA: ALA ratio	9.61	2.87	9.60	2.78
% Calories from Fat	33.68	5.71	35.29	7.48
% Calories from Carbohydrate	46.86	7.53	45.07	9.34
% Calories from Protein	18.65	5.57	19.20	5.48
% Calories from Alcohol *	0.82	2.01	0.43	1.55
% Calories from SFA	10.63	2.68	11.19	3.19
% Calories from MUFA	12.19	2.76	12.75	3.38
% Calories from PUFA	7.67	2.27	8.00	2.62
% Calories from n-3s	0.85	0.37	0.93	0.50
% Calories from LA	6.97	2.23	7.15	2.39

* Indicates statistically significant differences between groups at *p* = 0.05; *p*-values were not adjusted for multiple testing

**Table 3:** Multiple regression results for liver steatosis and fibrosis in a Mexican-origin Hispanic sample from Southern, Arizona (N = 285).

	Model 1 (Crude)			Model 2 ^a^			Model 3 ^b^		
	Estimate	*p*-Value	Adjusted R^2^	Estimate	*p*-Value	Adjusted R^2^	Estimate	*p*-Value	Adjusted R^2^
**Steatosis**									
LA:ALA Ratio	0.17	0.87	−0.003	0.20	0.83	0.183	-	-	
n-6:n-3 Ratio	−0.51	0.64	−0.003	−0.11	0.91	0.183	-	-	
**Fibrosis ^c^**									
LA:ALA Ratio	1.01	0.06	0.009	1.01	0.04	0.134	1.01	0.03	0.135
n-6:n-3 Ratio	1.02	0.03	0.014	1.02	0.01	0.143	1.02	0.01	0.145

**^a^** Model 2 adjusted for age, sex, BMI, and total energy intake. **^b^** Model 3 covariates were chosen by stepwise regression analysis (ending with sex and BMI in all models); Stepwise regression for steatosis yielded no significant covariates (such as the crude model); therefore, no model 3 results are presented for steatosis. **^c^** Fibrosis scores were log-transformed prior to analysis.

## Data Availability

No new data were created or analyzed in this study. Data sharing is not applicable to this article.
